# Broadband decoupling of intensity and polarization with vectorial Fourier metasurfaces

**DOI:** 10.1038/s41467-021-23908-0

**Published:** 2021-06-15

**Authors:** Qinghua Song, Arthur Baroni, Pin Chieh Wu, Sébastien Chenot, Virginie Brandli, Stéphane Vézian, Benjamin Damilano, Philippe de Mierry, Samira Khadir, Patrick Ferrand, Patrice Genevet

**Affiliations:** 1 Université Côte d’Azur, CNRS, CRHEA, Rue Bernard Gregory, Sophia Antipolis, Valbonne, France; 2 Aix Marseille Univ, CNRS, Centrale Marseille, Institut Fresnel, Marseille, France; 3 Department of Photonics, National Cheng Kung University, Tainan, Taiwan

**Keywords:** Metamaterials, Metamaterials

## Abstract

Intensity and polarization are two fundamental components of light. Independent control of them is of tremendous interest in many applications. In this paper, we propose a general vectorial encryption method, which enables arbitrary far-field light distribution with the local polarization, including orientations and ellipticities, decoupling intensity from polarization across a broad bandwidth using geometric phase metasurfaces. By revamping the well-known iterative Fourier transform algorithm, we propose “à la carte” design of far-field intensity and polarization distribution with vectorial Fourier metasurfaces. A series of non-conventional vectorial field distribution, mimicking cylindrical vector beams in the sense that they share the same intensity profile but with different polarization distribution and a speckled phase distribution, is demonstrated. Vectorial Fourier optical metasurfaces may enable important applications in the area of complex light beam generation, secure optical data storage, steganography and optical communications.

## Abstract (web summary)

Though multiplexing meta-holograms remains an attractive approach for realizing optical encoding, existing methods encode information based on the intensity of the holographic images. Here, the authors report vectorial metasurfaces that decouple and encode intensity and polarization information.

## Introduction

Optical waveform control plays a critical role in the optical systems for various applications. Among the different methods to address the electromagnetic field distribution in the far field, optical metasurfaces^1–12^—artificial materials that consist of subwavelength structure arrays—are capable of tailoring the waveform of the electromagnetic waves with an unpreceded level of precision. In particular, due to the versatility of this approach, it is possible to engineer both amplitude and polarization information at will. Vectorial meta-holograms with arbitrary polarization have been developed using a diatomic reflective metasurface^13,14^ and a geometric phase-based metasurface^15^. However, the generated polarization is limited by the multiplexing metasurface’s sub-pixels, which are not able to realize arbitrary spatially distributed polarization as yet. Some efforts have been made by combining geometric phase and propagation phase^16,17^, but it severely suffers from narrow bandwidth. A broadband wavefront control that can decouple amplitude from polarization information has yet to be demonstrated.

One of the most important applications of meta-holograms is information security, which is important in many areas of the society, such as protecting individuals, industries, and military information from leaks and stealing. Among the different communication channels and information sharing techniques, photonics is the most efficient and effective way of carrying information across long distances. Optical waveforms possess many degrees of freedom, such as amplitude, phase, frequency, and polarization, and each can be used for data encoding. Moreover, optical encoding methods require specific professional equipment for data encoding, providing a more secure way towards high-security information encoding. Various optical encoding methods have been developed based on the intensity, such as spatial correlators^18^, optical exclusive or (XOR) image encryption^19^, phase-shifting interferometry^20^, polarization-dependent images^21,22^, Lippmann plate^23^, and holograms^24,25^. Many other efforts of optical encoding have been made by using multiplexing meta-hologram that can encode the optical information into multi-channels of holographic images^26–30^. The most common approach is based on polarization-dependent meta-hologram, which creates different holographic images using different polarizations of the incident beam^31–36^. Chiral meta-holograms are also introduced for direction-dependent holographic encoding^37–40^. Other encoding methods relying on incident wavelength^41^, nonlinear effect^42^, spatial frequency^43^, orbital angular momentum^44–46^, and tunable metasurface^47–49^ are also demonstrated. It is noteworthy that all of these proposed multiplexing meta-holograms encode information on the intensity of the holographic images.

In this study, we propose a vectorial Fourier metasurface for which amplitude and polarization information can be addressed independently one from the other. We utilize this specificity to encode intensity and two polarization information channels, namely ellipticity and azimuth, to produce far-field decoupled images. The image refers to the spatial distribution of either total intensity, ellipticity, or azimuth information. The design of the metasurface is realized using a modified iterative Fourier transform (IFT) algorithm that does not only consider far-field amplitude but also the far-field polarization spatial distribution. Our calculation results consist of discretized transmission matrices representing the spatial amplitude and polarization into pixelated profiles. The design of the nanostructured interfaces capable of matching these distributions therefore requires precise control of all properties of transmitted light. To this end, we composed arbitrary amplitude, phase, and polarization pixels by superposing two amplitude-modulated and phase-delayed beams with the opposite circular polarizations (CPs).

With respect to previously proposed methods, our approach defines the metasurface capability by considering the polarization distribution in the far field only, i.e., as a result of the propagation leading to left CP (LCP) and right CP (RCP) far fields. As the metasurface plane is encoded via the Fourier transformation of targeted fields, the realization of optical information encoding in this work is completely outperforming than that of previous demonstrations with multiplexing^15^. As a proof of principle, we designed a series of far-field intensity profiles presenting a given donut-like intensity distribution structured with different polarization orientations. Interestingly, these vectorial fields look like the well-known cylindrical vector beams (CVBs; previously discussed in the literature). Nevertheless, they differ strongly to CVB in the sense that, even if the polarization is maintained, their long-range far-field phase distribution is lost during the optimization process. Roughly speaking, the randomization of the far-field phase using IFT techniques produces granular intensity distributions or intensity-modulated speckle signals, which match the overall targeted CVB donut intensity and polarization. These field profiles are extremely interesting, as they are mixing long-range transverse coherence (correlation between the fields at different points) with very short-range spatial phase correlation (the degree to which the spatial phase are related). In addition, we demonstrate a class of optical interfaces that encodes the orientation angle and ellipticity angle of the polarization in a uniformly distributed intensity profile. To resolve the encoded information, we use both conventional Stokes parameter measurements and vectorial ptychography to characterize both metasurface and their far-field complex amplitudes.

## Results

### Design method

**Fig. 1:**
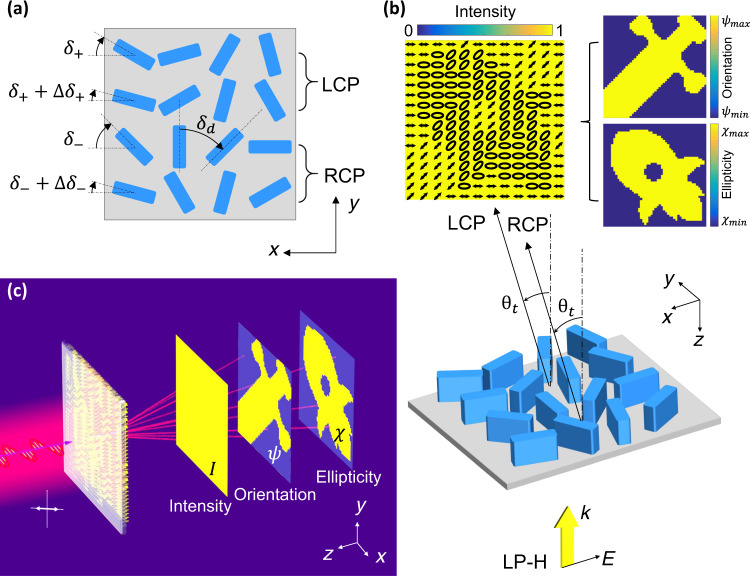
Design principle of vectorial Fourier metasurface for arbitrary far-field light distribution of intensity and polarization. **a** Top view of one pixel of the metasurface. Arbitrary polarization requires superposition of two orthogonal polarizations (chosen here as RCP and LCP) with controllable relative amplitudes and phases. To do so, each CP state is produced by two lines of the same handiness with different SOA of $\triangle {\delta }_{\pm }$ to control the relative amplitude and ${\delta }_{\pm }$ to control the relative phase. **b** Perspective view of the metasurface and far-field light distribution. The LP input light can be decomposed into two CP beams, which are deflected to the same angle of ${\theta }_{\mathrm{t}}$. The holographic phase information is encoded in the LCP and RCP independently, so that arbitrary polarization is realized by the superposition of the two CP beams. **c** Schematic of the intensity and polarization decoupling using vectorial Fourier metasurface. The orientation angle and ellipticity of the polarization exhibiting a “Blade” and a “Rocket” images are encoded in a uniformly distributed intensity profile.

The design principle of the vectorial Fourier metasurface is shown in Fig. 1. Each pixel of the metasurface consists of four lines of phase gradient supercells as shown in Fig. 1a, in which the top two lines and bottom two lines of meta-structures are arranged counter-clockwise and clockwise, respectively, with the same orientation increment angle of ${\delta }_{\mathrm{d}}$. Each building block of the pixels, the pillar meta-structure, acts as a half-waveplate that converts the handiness of the incident CP beams and imposes a geometry phase (also called Pancharatnam–Berry (PB) phase) of $\pm 2\delta$, where $\delta$ is the orientation angle of each pillar (the signs “−” and “+” denoted clockwise and counter-clockwise rotation, respectively), i.e., $|+\rangle \to {e}^{i2\delta }|-\rangle$ and $|-\rangle \to {e}^{-i2\delta }|+\rangle$, where $|+\rangle$ represents LCP and $|-\rangle$ represents RCP. Considering that the incident linear polarized (LP) light can be decomposed into LCP and RCP, the clockwise lines in a pixel deflect the LCP light to RCP light with a deflection angle of ${\theta }_{\mathrm{t}}={\rm{arcsin }}\left(\frac{2{\delta }_{\mathrm{d}}}{{k}_{0}P}\right)$ as shown in Fig. 1b, where ${k}_{0}$ is the wavenumber in the free space and *P* is the period of the unit cell. The counter-clockwise lines in the same pixel deflect the RCP to LCP at the same angle of ${\theta }_{\mathrm{t}}$. The starting orientation angle of the four lines from top to bottom are ${\delta }_{+}$, ${\delta }_{+}+\triangle {\delta }_{+}$, ${\delta }_{-}$, and ${\delta }_{-}+\triangle {\delta }_{-}$, where $\triangle {\delta }_{\pm }$ and ${\delta }_{\pm }$ are respectively used to control the relative amplitude and phase between LCP and RCP. We ignore the co-polarization in the following text, simply because it is diffracted at the zero order and it does not interfere with the cross-polarized fields. The complex amplitude ${a}^{m}$ in the metasurface plane is given by,

1 $${a}^{m}\left({x}^{m},{y}^{m}\right)={A}_{+}^{m}\left({x}^{m},{y}^{m}\right){e}^{i{\varphi }_{+}^{m}\left({x}^{m},{y}^{m}\right)}+{A}_{-}^{m}\left({x}^{m},{y}^{m}\right){e}^{i{\varphi }_{-}^{m}\left({x}^{m},{y}^{m}\right)}$$

where the superscript *m* represents the metasurface plane, ${x}^{m}$ and ${y}^{m}$ represent the pixel positions in the metasurface plane, and ${A}_{\pm }^{m}\left({x}^{m},{y}^{m}\right)$ and ${\varphi }_{\pm }^{m}\left({x}^{m},{y}^{m}\right)$ are the amplitude and phase of pixel (${x}^{m},{y}^{m}$) at the metasurface plane generated by the two CP of the light beam. For simplicity, in the following we ignore the notation of (${x}^{m},{y}^{m}$). The amplitude ${A}_{\pm }^{m}$ is controlled by the rotation angle difference of $\triangle {\delta }_{\pm }$ due to the interference between two lines of LCP (or RCP) as,

2 $${A}_{\sigma }^{m}=\left|{e}^{-i2\sigma {\delta }_{\sigma }}+{e}^{-i2\sigma \left({\delta }_{\sigma }+\triangle {\delta }_{\sigma }\right)}\right|/2=\sqrt{\left(1+{\rm{cos }}2\triangle {\delta }_{\sigma }\right)/2}$$

where $\sigma =+$ (or $+1$) represents LCP and $\sigma =-$(or $-1$) represents RCP. The phase ${\varphi }_{\pm }^{m}$ is generated by the rotation angle of ${\delta }_{\pm }$, thanks to the geometric phase as,

3 $${\varphi }_{\sigma }^{m}=-\,2\sigma {\delta }_{\sigma }$$

Therefore, by varying the value of ${\delta }_{\pm }$ and $\triangle {\delta }_{\pm }$, arbitrary amplitude and phase information in the metasurface plane can be assigned to each pixel independently from the others, so as to control far-field amplitude and polarization information at will.

**Fig. 2:**
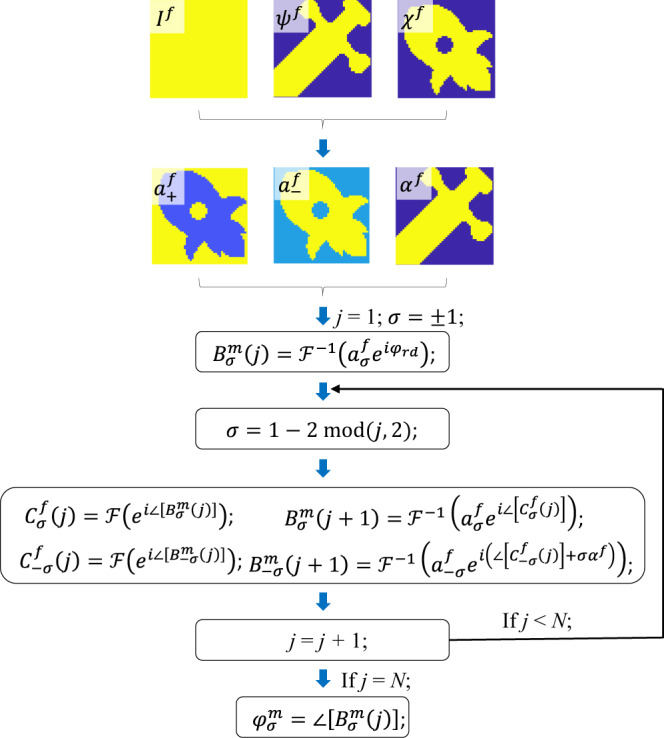
Modified iterative Fourier transform algorithm. With respect to intensity-only iterative Fourier transform algorithm, the current version considers several input information to realize diffraction patterns with arbitrary intensity, azimuth, and ellipticity angles of the polarization. The algorithm converges to a vectorial profile optimizing the amplitude of both LCP (${a}_{+}^{f}$) and RCP (${a}_{-}^{f}$), and the phase difference between the two CP beams (${\alpha }^{f}$). The notation $\sigma$ represents the handiness of the CP beam, where $+$ or $+1$ represents LCP and $-$ or $-1$ represents RCP. A random phase of ${\varphi }_{\mathrm{rd}}$ is used for the starting phase. The number of iterations is *N* = 100. The final holographic phase of the metasurface is ${\varphi }_{\sigma }^{m}$. The superscript *m* indicates the metasurface plane and *f* is the image plane in the far field (see more details in Supplementary Note 1).

To decouple amplitude from far-field polarization information, we propose modifying the conventional Gerchberg–Saxton (GS) algorithm to a version working for vectorial fields. The GS utilizes IFT as shown in Fig. 2 (see more details in Supplementary Note 1)^17,26,27^, and in its vectorial version—instead of converging to a phase profile in the metasurface plane—we consider the phase profiles of two CP beams noted ${\varphi }_{\sigma }^{m}$ realized by rotating the angle of ${\delta }_{\sigma }$ according to Eq. 3. In this implementation, the far-field polarization can be controlled over the entire profile, despite the fact that GS converges to designs with randomly distributed far-field phase profile. The condition for far-field polarization addressing requires that the phase retardation between orthogonal polarization channels is properly adjusted, i.e., the phase value for both polarization channels is randomly distributed on the transverse plane with a controllable phase retardation.

For a convenience purpose, we keep the amplitude in the metasurface plane ${A}_{\pm }^{m}$ uniform for all pixels, i.e., $\triangle {\delta }_{+}$ and $\triangle {\delta }_{-}$ are two constant values for all pixels determined by the total intensity of two CP beams ${I}_{\pm }^{m}$. The latter are calculated considering the intensity integral of all pixels in the image plane as: ${I}_{\pm }^{m} = {I}_{\pm }^{f} = \mathop{\sum}\nolimits_{x, y=1}^{{N_{x}, N_{y}}} {\left(a_{\pm }^{f}{\left(x^{f}, y^{f}\right)}\right)}^{2}$, where the superscript *f* represents the far-field image plane, (${x}^{f},$ ${y}^{f}$) represent the pixel position in the far-field image plane, *N*_*x*_ × *N*_*y*_ is the total pixel number, and ${a}_{\pm }^{f}\left({x}^{f},{y}^{f}\right)$ are the amplitude of LCP and RCP light of each pixel $\left({x}^{f},{y}^{f}\right)$. It can be shown that the rotation angles $\triangle {\delta }_{\pm }$ are given by (see more details in Supplementary Note 2),

4 $$\left\{\begin{array}{cc}\triangle {\delta }_{\sigma }=0,\hfill& {if}\; {I}_{\sigma }^{f}\;\ge\; {I}_{-\sigma }^{f}\\ \triangle {\delta }_{\sigma }={\rm{acos}}\left(\frac{2\mathop{\sum }\limits_{x,y=1}^{{N}_{x},{N}_{y}}{\left({a}_{\sigma }^{f}\left({x}^{f},{y}^{f}\right)\right)}^{2}}{\mathop{\sum }\limits_{x,y=1}^{{N}_{x},{N}_{y}}{\left({a}_{-\sigma }^{f}\left({x}^{f},{y}^{f}\right)\right)}^{2}}-1\right)/2,& {if}\; {I}_{\sigma }^{f} \;<\; {I}_{-\sigma }^{f}\end{array}\right.$$

Equations (3) and (4) are then used to recover the orientation angles of each pixel of the metasurface.

**Fig. 3:**
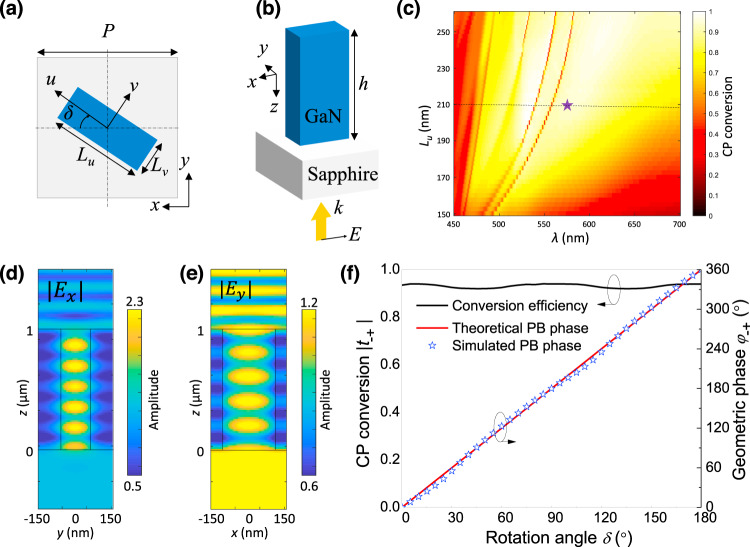
Simulated results of metasurface with birefringent GaN nanopillars on sapphire substrate with dimension of *P* = 300 nm and *h* = 1 μm. **a** Top view and **b** perspective view of one meta-structure. The numerical calculation is performed on unit cell of GaN nanopillar on sapphire substrate considering periodic boundary conditions on *x* and *y*. The width of the GaN nanopillar is fixed as *L*_v_ = 120 nm. **c** The simulated CP conversion by sweeping the length of the GaN nanopillar *L*_u_ from 150 to 260 nm in the wavelength range from 450 to 700 nm with zero rotation angle. The dash line represents the length of *L*_u_ = 210 nm. The purple star represents the wavelength at $\lambda$ = 575 nm, which is the chosen point in **d**–**f**. **d** The simulated electric field distribution *E*_*x*_ at the plane of *x* = 0 and **e** *E*_*y*_ at the plane of *y* = 0. **f** By rotating the GaN nanopillars with an angle of *δ* from 0 to 180°, a near-unity CP conversion is shown in the black curve and a geometric phase from 0 to 360° is obtained. The simulated results are represented by the blue star, in very good agreement with the expected PB phase calculation (red curve).

The meta-structures are simulated using full-wave finite-difference time-domain (FDTD) and the simulation results are shown in Fig. 3. The top view and perspective view of one meta-structure are shown in Fig. 3a, b, respectively. One-micrometer-tall GaN nanopillars, grown on low-index lattice-matched Sapphire substrate, are realized with rectangular cross-sections to induce structural birefringence. Both GaN and sapphire are transparent in the entire visible range, which are perfect candidates for the design of visible optical metasurfaces. The period of the nanostructure unit cell is *P* = 300 nm to avoid spurious diffraction effects in the substrate. The width is fixed to *L*_v_ = 120 nm. The CP conversion efficiency is shown in Fig. 3c with the long axis of the nanopillar *L*_u_ swept from 160 to 260 nm and the wavelength *λ* swept from 450 to 700 nm. The dash line indicates the CP conversion at *L*_u_ = 210 nm, where the CP conversion efficiency is higher than 50% across almost the entire visible range. Figure 3d, e show the electric field distribution along short and long axis of the pillar, respectively, at the point of *L*_v_ = 120 nm and *λ* = 575 nm (the purple star in Fig. 3c). It is shown that there are 5.5 and 5 oscillations of electric field in *E*_*x*_ and *E*_*y*_ in the GaN nanopillar, i.e., a signature of half a wavelength retardation difference, which verifies that the meta-structure acts as a nanoscale half-waveplate for these structural parameters and operation wavelength. In addition, when the nanopillar is rotated with an angle of *δ*, a geometric phase of 2*δ* is obtained on cross-CP as shown in Fig. 3f. The simulated geometric phase by using FDTD shown in blue stars agrees well with the theoretical one (${\varphi }_{\mathrm{RL}}=2\delta$) shown by the red curve. In addition, the CP conversion efficiency between LCP and RCP is, as expected, near unity as shown by the black curve.

**Fig. 4:**
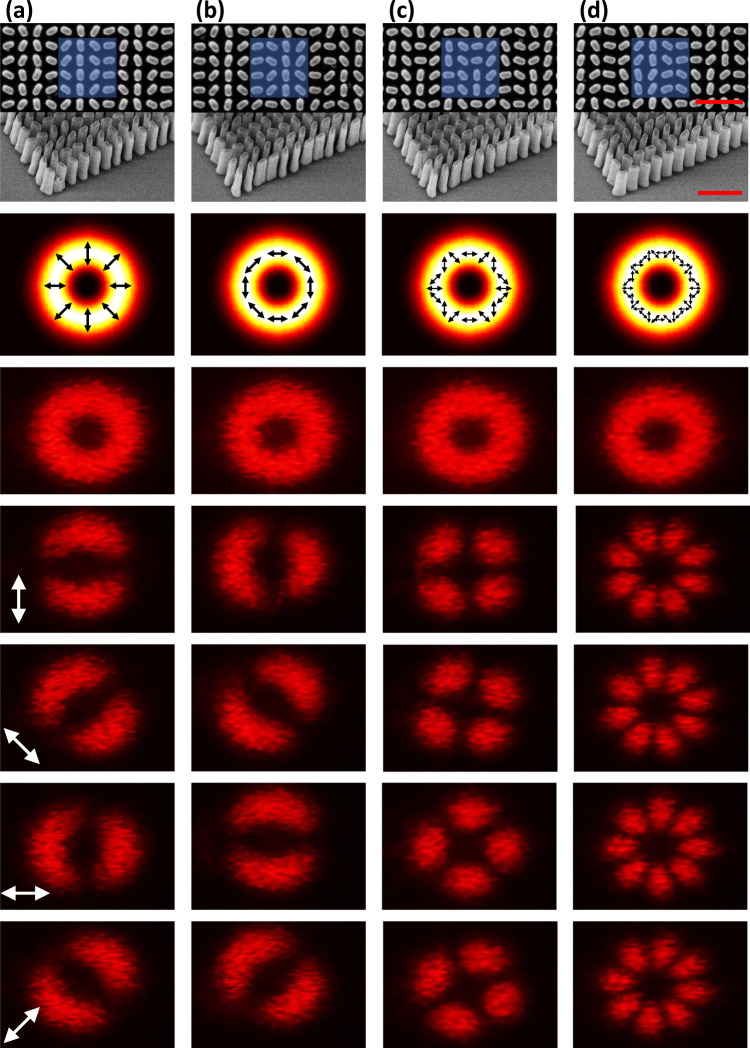
Fabricated results and optical measurement of the Stokes polarization parameters of the donut polarization-distributed field profile. Field distribution with **a** *l* = 1, *m* = 0; **b** *l* = 1, *m* = 1; **c** *l* = −2, *m* = 0; **d** *l* = −4, *m* = 0. Top row: fabricated results with the top view (top panels) and tilt view (bottom panels). The blue area shows one pixel of the meta-hologram. The red scale bar represents 1 μm. Second row: intensity profiles of the designed vectorial fields. The black arrow represents the local polarization. Third row: measured intensity profiles of the field distributions without linear polarizer. Fourth to seventh rows: measured intensity profiles of the field distributions with a linear polarizer. The white arrow represents the transmission axis of the linear polarizer.

**Fig. 5:**
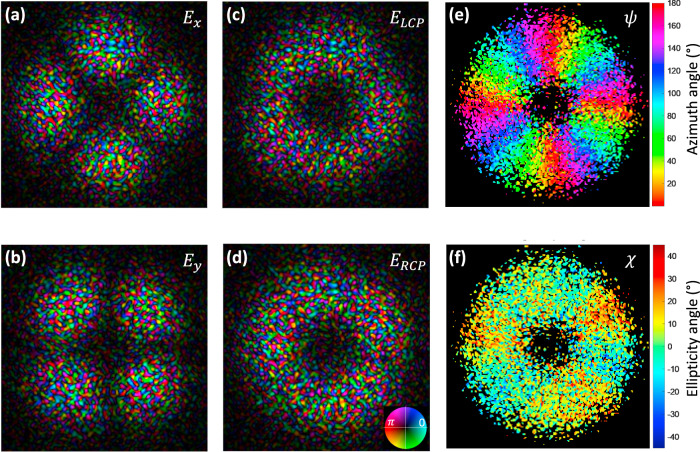
Complex amplitude and polarization information of far field as retrieved by the ptychographic measurement. Measured complex amplitude of **a** *x*-pol., **b** *y*-pol., **c** LCP, and **d** RCP components. The phase profiles for the four polarization components show typical speckle phase distributions. The inset figure in **d** is the color bar with phase encoded as hue and amplitude as brightness. Measured **e** azimuth angle and **f** ellipticity angle of polarization information.

**Fig. 6:**
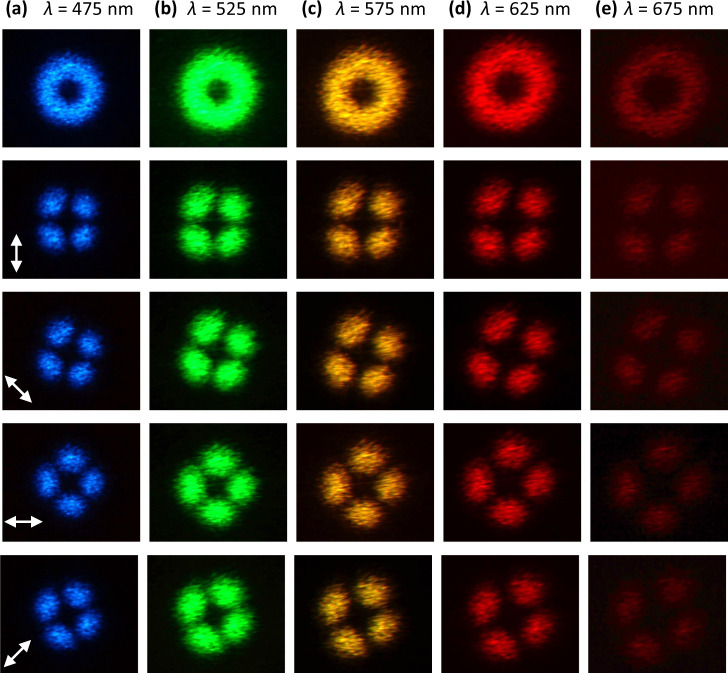
Broadband characterization of the vectorial metasurfaces designed for a field profiles with *l* = −2, *m* = 0. The measured intensity profiles at different wavelengths of **a** *λ* = 475 nm, **b** *λ* = 525 nm, **c** *λ* = 575 nm, **d** *λ* = 625 nm, **e** *λ* = 675 nm. Top row: measured intensity profiles without linear polarizer. Second to fifth rows: measured intensity profiles with a linear polarizer. The white arrow represents the transmission axis of the linear polarizer.

In order to validate our approach to decouple intensity from polarization, we conceived a series of spatially variant far-field polarization profiles distributed on a donut far-field intensity profile (with the same radius), but having different azimuthal angle of the linear polarization defined by $\psi \left(x,y\right)=l\varphi \left(x,y\right)+m\pi /2$, where *l* is an integer number that represents the turns of the polarization rotation encircling the donut intensity profile, *x* and *y* are the coordinate of beam, $\varphi \left(x,y\right)={{\rm{tan }}}^{-1}(\frac{y}{x})$, *m* = 0 represents the radial mode, and *m* = 1 represents the azimuthal mode. We demonstrated four designs with different combinations of *m* and *l* (see more details in Supplementary Fig. 3). The scanning electron microscope images of the fabricated vectorial beam metasurfaces are shown in the first row of Fig. 4. The second row represents the designed intensity and polarization profiles. The measured total intensity profiles are shown in the third row, which agree well to the designed intensities. By placing a linear polarizer with different rotation angles in front of the vectorial beams, different patterns are observed from the fourth to the seventh rows. Interestingly, even if these beams resemble the well-known CVBs, we prove that they do not feature long-range spatial phase correlation. To do so, the amplitude and polarization information of the vectorial far-field patterns were also investigated by means of vectorial ptychography. This computational microscopy technique can indeed provide a quantitative map of the metasurface, thanks to the retrieval of its Jones matrix^50^, mapped at a microscopic resolution of the whole sample area (see “Methods” and Supplementary Fig. 2b). This quantitative knowledge of the metasurface optical properties makes it possible to model totally the far-field patter, by modeling a horizontally polarized illumination on the sample, followed by propagation (see Supplementary Fig. 2c). The resulting far-field complex amplitude distributions, either on a LP or on an RCP/LCP decomposition basis, as shown in Fig. 5, exhibit a short spatial phase correlation with a clear speckle patter, while maintaining polarization over the whole intensity pattern. Indeed, with respect to CV beams that are vectorial solutions of Maxwell’s equations obeying axial symmetry in both amplitude and phase, our solution to produce spatially distributed amplitude and polarization field does not impose long-range spatial phase correlation. These fields could be beneficial for practical applications in laser machining, remote sensing, and so forth^51,52^, or to decouple phase and polarization in singular optics. Using PB phase-tuning mechanisms, the polarization encoding is simply given by the rotation angle of the nanostructures, resulting in a broad operating bandwidth. It reveals that broad operating bandwidth is generally not achievable with a combination of propagation and PB phases^16^. The characterization of the broadband properties is shown in Fig. 6. A CV beam with *l* = −2 and *m* = 0 is measured from *λ* = 475 nm to *λ* = 675 nm. A donut intensity profile is shown in the first row without polarizer. Subsequently, we insert a linear polarizer in front of the image. The same pattern is observed for all of the wavelength with fixed transmission axis of polarizer as shown from the second to fifth rows, indicating that the metasurface could maintain polarization distribution properties over a broad wavelength range. The efficiency of the CV beam is in the range of 5 ~ 17% across the entire visible range as shown in Supplementary Fig. 5, which is a bit low due to the fabrication errors and large pixel size. The latter is larger than the operating wavelength, resulting in higher-order images, which decreases the efficiency of the interested order.

After verification of the design approach with simple vectorial beam, we propose to encode optical information relying on the azimuth and ellipticity angles of the polarization information rather than encoding intensity profiles of conventional polarization states, realizing a sort of holographic polarization-only encoding technique. Two meta-holograms with the same intensity profile but different azimuth and ellipticity angles of the polarization are designed as shown in the Supplementary Fig. 6. The fabricated results of the two metasurfaces are shown in Supplementary Fig. 7. As the information is only encoded on the polarization properties, i.e., spatial distribution of the orientation and ellipticity, an additional retrieval method based on local Stokes polarimetry is required^53,54^. Stokes parameters, which include the optical quantities of interest, are generally obtained using two sets of measurements, cascading a waveplate with the phase difference between fast axis and slow axis of $\phi$, and a linear polarizer with rotation angle of $\theta$ with respect to the *x*-axis in front of the image. The measured intensity profiles after the cascaded waveplate and linear polarizer are related to $\phi$ and $\theta$, which are denoted as $I\left(\theta ,\phi \right)$. Therefore, the measured azimuth and ellipticity angles of the polarization are described as (see more details in Supplementary Note 3),

5 $$\psi =\frac{1}{2}{{\rm{tan }}}^{-1}\left(\frac{2I\left(45^\circ ,0^\circ \right)-I\left(0^\circ ,0^\circ \right)-I\left(90^\circ ,0^\circ \right)}{I\left(0^\circ ,0^\circ \right)-I\left(90^\circ ,0^\circ \right)}\right)\left(-\frac{\pi }{2} \;<\; \psi \le \frac{\pi }{2}\right)$$

6 $$\chi =\frac{1}{2}{{\rm{sin }}}^{-1}\left(\frac{I\left(0^\circ ,0^\circ \right)+I\left(90^\circ ,0^\circ \right)-2I\left(45^\circ ,90^\circ \right)}{I\left(0^\circ ,0^\circ \right)+I\left(90^\circ ,0^\circ \right)}\right)\left(-\frac{\pi }{4} \;<\; \chi \le \frac{\pi }{4}\right)$$

**Fig. 7:**
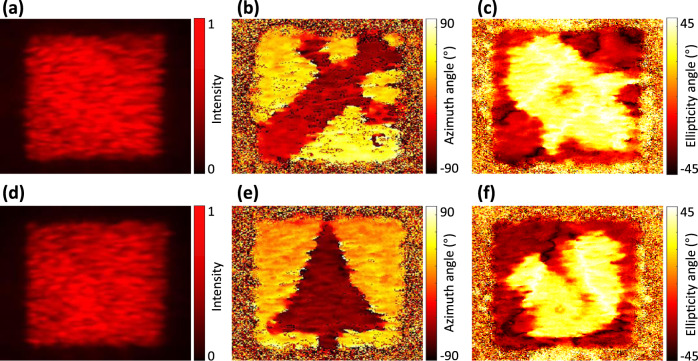
Measurement results of vectorial encoded images. **a**–**c** Show the data obtained for the first metasurface design. **d**–**f** Correspond to the data for the second metasurface design. **a**–**f** Measurement results using conventional optical setup to image the Stokes parameters. **a**, **d** Measured intensity; **b**, **e** azimuth angle; **c**, **f** ellipticity angle of the polarization. Both interfaces encode a similar uniformly distributed intensity profile as shown in **a** and **d**. Color-coded images displaying the ellipticity and the orientation; images reveal a “Blade,” a “Rocket,” a “Tree,” and a “Squirrel” encoded polarization images as shown in **b**, **c**, **e**, and **f**, respectively.

In additional to these measurements, the total intensity profiles, i.e., with spatially varying polarization distribution, are measured directly, without any waveplate and/or polarizer. The measured results of the first metasurface are shown in Fig. 7a–c. As expected from the design, a uniform intensity profile is observed in Fig. 7a. However, both a “Blade” and a “Rocket” images are shown when looking at the polarization spatial distribution in the azimuth and ellipticity angles, respectively. Another design with the same uniform intensity profile but a “Tree” and a “Squirrel” polarization information is obtained in Fig. 7d–f. Besides, we also used vectorial ptychography to map both the amplitude and polarization information of the vectorial far-field patterns (see more details in “Methods” and Supplementary Fig. 2b). The Jones matrix maps of the metasurface as retrieved by vectorial ptychography are shown in Supplementary Fig. 8, whereas the far field is shown in Supplementary Fig. 9, including intensity and polarization information. As expected, a uniformly distributed intensity profile is observed in both designs of Supplementary Fig. 9a, d. Moreover, in the azimuth angle and ellipticity angle of polarization map, images of “Blade” and “Rocket” are respectively obtained in Supplementary Fig. 9b, c and images of “Tree” and “Squirrel” are observed in Supplementary Fig. 9e, f.

## Discussion

In conclusion, we have demonstrated a general method to design vectorial Fourier metasurfaces, which decouple intensity from polarization information, such that spatially distributed full polarization profiles with arbitrary intensity distribution can be realized. The vectorial Fourier metasurfaces are conceived using a modified IFT algorithm that optimizes the transmission information properties to encode simultaneously both intensity and polarization far-field distribution. We produce an interesting series of far-field beam profile with donut-like intensity and spatially distributed polarization, resembling CVBs, but with randomly distributed far-field phase distribution. To fully characterize the optical response of our Fourier metasurfaces, we retrieve the complete Jones matrix of the metasurface using vectorial ptychography and proved the short-range phase correlation in contrast to the long-range polarization distribution, indicating that both polarization channels have spatially correlated phase profiles. Furthermore, the proposed vectorial Fourier metasurfaces are able to encode complex polarization information onto uniform distributed intensity profiles. We demonstrated that a “Blade” (or “Tree”) and a “Rocket” (or “Squirrel”) images can be multiplexed and separately decrypted from the orientation angle and ellipticity angle of the polarization on a uniformly distributed intensity profile of a holographic image. Vectorial Fourier encoding could highly enhance the information security, having various promising applications in data encryption, optical ID tags for authentication and verification, and high-density optical data storage, but also for specific applications including optical trapping and laser machining.

## Methods

### Device fabrication

A GaN thin film with 1 μm thickness is grown on a double-side polished c-plane sapphire substrate using molecular beam epitaxy RIBER system. Conventional electron beam lithography processes are used for the etching of GaN nanopillars. The poly (methyl methacrylate) resist (495A4) with ~180 nm is spin coated on the GaN and baked on a hot plate with temperature of 125 °C. It is exposed with designed patterns at 20 keV (Raith ElphyPlus, Zeiss Supra 40) and developed in 3 : 1 IPA : MIBK solution. Oxygen plasma etching (reactive ion etching (RIE), Oxford System) is used to clean the residual resist that is not completely removed during development. The sample is deposited with a 50 nm thickness of Nickel using E-beam evaporation and is immersed into acetone solution for lift-off process to obtain the Nickel hard mask. By using RIE (Oxford System) with a plasma composed of Cl_2_CH_4_Ar gases, the pattern is transferred to the GaN. The residual nickel hard mask is removed by chemical etching with 1 : 1 H_2_O_2_ : H_2_SO_4_ solution, revealing the GaN nanopillars. See more details of fabrication processes in Supplementary Fig. 1.

### Conventional optical setup

The optical setup for characterizing the projected far-field is shown in Supplementary Fig. 2a. A laser beam propagates through a linear polarizer and a quarter waveplate (QWP). In order to avoid the birefringent effect of the sapphire substrate, we calibrate the polarization of the input light with a bare sapphire substrate to make sure the input light is LP in horizontal direction by controlling the rotation angle of the previous linear polarizer and QWP. After an achromatic lens with a focal length of 50 mm, the laser beam is weakly focused on the metasurfaces. The first-order holographic image is projected onto a projector placed 10 cm away from the metasurface. A selected QWP with fast axis at the horizontal and a linear polarizer with axis of transmission at angle $\theta$ are used to analyze the images and measure the Stokes parameters.

### Optical vectorial ptychography

Measurements were carried on a custom setup, based on an inverted microscope equipped with a motorized stage operating at 635 nm^50,55^. The measurement principle, which involves the recording of a series of far-field intensity patterns while scanning the specimen under a coherent illumination, is schematized in Supplementary Fig. 2b. All reported measurements in this work were obtained under the following specific parameters: the illumination probe was reduced to an effective diameter of 50 μm, by placing a 2 mm diameter iris diaphragm in the image plane of a ×40 objective lens (ACHN-P, NA 0.65, Olympus). The camera (Stingray F-145B, Allied Vision, 320 × 240 effective pixels of 25.8 × 25.8 μm^2^ after binning) was placed 190 mm after the diaphragm. Measurement scan was repeated for nine combinations of LP illumination (0°, 60°, and 120°) and analysis (0°, 60°, and 120°). Data processing was performed by means of a dedicated conjugate gradient algorithm^56^ running on a multi-graphics processor unit (DGX Station, NVIDIA), allowing the estimation of Jones matrices of the meta-holograms. The vectorial far field was obtained by modeling an LP illumination of the metasurface, followed by a propagation by fast Fourier transform of the vectorial exit field, as illustrated in Supplementary Fig. 2c. The spatial distribution of the propagation of the CV beams and two metasurface encryption are shown in Supplementary Movies 1–3.

## Supplementary information

Description of Additional Supplementary Files

Supplementary Information

Supplementary Data 1

Supplementary Movie 1

Supplementary Movie 2

Supplementary Movie 3

## Data Availability

Ptychography raw data and other data that support the findings are available upon request to the authors.

## Code availability

The code used for the vectorial meta-hologram design is available in the Supplementary Note 4 and Supplementary Data 1.
