# Supplementary material for: Broadband decoupling of intensity and polarization with vectorial Fourier metasurfaces
Source: Nat Commun. 2021 Jun 15;12:3631. doi: 10.1038/s41467-021-23908-0 (PMC8206126; doi:10.1038/s41467-021-23908-0)
Supplement: Supplementary file 1 — Description of Additional Supplementary Files [file 41467_2021_23908_MOESM1_ESM.pdf]

Description of Additional Supplementary Files

File Name: Supplementary Data 1
Description: Modified_GS_Algorithm

File Name: Supplementary Movie 1
Description: Propagation of CV beams

File Name: Supplementary Movie 2
Description: Propagation of metasurface for “Blade” and “Rocked” encryption

File Name: Supplementary Movie 3
Description: Propagation of metasurface for “Tree” and “Squirrel” encryption
