# Supplementary material for: Broadband decoupling of intensity and polarization with vectorial Fourier metasurfaces
Source: Nat Commun. 2021 Jun 15;12:3631. doi: 10.1038/s41467-021-23908-0 (PMC8206126; doi:10.1038/s41467-021-23908-0)
Supplement: Supplementary file 2 — Supplementary Information [file 41467_2021_23908_MOESM2_ESM.pdf]

Supplementary Information

Broadband Decoupling of Intensity and Polarization with Vectorial Fourier Metasurfaces

Qinghua Song[1], Arthur Baroni[2], Pin Chieh Wu[3], Sébastien Chenot[1], Virginie Brandli[1], Stéphane Vézian[1], Benjamin Damilano[1], Philippe de Mierry[1], Samira Khadir[1], Patrick Ferrand[2] and Patrice Genevet[1†]

[1] *Université Côte d'Azur, CNRS, CRHEA, Rue Bernard Gregory, Sophia Antipolis 06560 Valbonne, France*

[2] *Aix Marseille univ, CNRS, Centrale Marseille, Institut Fresnel, 13013 Marseille, France*

[3] *Department of Photonics, National Cheng Kung University, Tainan 70101, Taiwan*

[†] *Corresponding Author: Patrice.Genevet@crhea.cnrs.fr*

Supplementary Note 1: Modified iterative Fourier transform algorithm

In order to decouple the amplitude and polarization information, a modified iterative Fourier transform algorithm is used to calculate the holographic phase profile for both LCP and RCP as shown in Figure 2 in the main text. Assuming an arbitrary far field information with intensity I^f, azimuth angle ψ^f and ellipticity angle χ^f (the superscript *f* represents the far field image plane, *m* represents the metasurface plane), it can be converted to the amplitude information for LCP (a_+^f) and RCP (a_-^f), and phase difference between LCP and RCP (α^f) as shown below,

$$a_\sigma^f = \sqrt{(I - \sigma I \sin(2\chi))/2} \quad (1)$$

$$\alpha^f = 2\psi^f \quad (2)$$

where $\sigma = +$ (or $+1$) represents LCP, $\sigma = -$ (or -1) represent RCP. We apply a random phase φ_{rd} into the amplitude information of Supplementary Eq. 1 to obtain the initial complex amplitude $a_\sigma^f e^{i\varphi_{rd}}$ and perform the inverse Fourier transform to get the initial metasurface information,

$$B_\sigma^m(1) = \mathcal{F}^{-1}\left(a_\sigma^f e^{i\varphi_{rd}}\right) \quad (3)$$

where $\mathcal{F}$ and $\mathcal{F}^{-1}$ are the operators for Fourier transform and inverse Fourier transform, respectively. Considering the amplitude in the metasurface plane for LCP and RCP is constant, an iterative Fourier transform process is used to get the phase information as following. The iteration number *j* is an integer number starting from 1. If *j* is an odd number, the algorithm is described as,

$$C_-^f(j) = \mathcal{F}\left(e^{i\angle[B_-^m(j)]}\right) \quad (4)$$

$$B_-^m(j+1) = \mathcal{F}^{-1}\left(a_-^f e^{i\angle\left[C_-^f(j)\right]}\right) \quad (5)$$

$$C_+^f(j) = \mathcal{F}\left(e^{i\angle[B_+^m(j)]}\right) \tag{6}$$

$$B_+^m(j+1) = \mathcal{F}^{-1}\left(a_-^f e^{i\left(\angle\left[C_-^f(j)\right]-\alpha^f\right)}\right) \tag{7}$$

where $\angle[X]$ represent the phase of complex number X. If j is an even number, the algorithm becomes,

$$C_+^f(j) = \mathcal{F}\left(e^{i\angle[B_+^m(j)]}\right) \tag{8}$$

$$B_+^m(j+1) = \mathcal{F}^{-1}\left(a_+^f e^{i\angle\left[C_+^f(j)\right]}\right) \tag{9}$$

$$C_-^f(j) = \mathcal{F}\left(e^{i\angle[B_-^m(j)]}\right) \tag{10}$$

$$B_-^m(j+1) = \mathcal{F}^{-1}\left(a_+^f e^{i\left(\angle\left[C_+^f(j)\right]+\alpha^f\right)}\right) \tag{11}$$

When the iteration number j reaches a given value N, the iteration process ends. The final phase information of the metasurface is given as,

$$\varphi_\sigma^m = \angle[B_\sigma^m(j)] \tag{12}$$

Supplementary Eq. 4-12 can be simplified as Figure 2 in the main text.

Supplementary Note 2: Determination of rotation angle of meta-structures

Since the meta-structure with a rotation angle of δ converts the CP light of $|\pm\rangle$ to $|\mp\rangle$ with geometric phase of $\pm 2\delta$ that is used to encode the holographic phase information, the rotation angle of the meta-structure is given as,

$$\delta_\sigma = -\sigma\varphi_\sigma^m/2 \tag{13}$$

Since the intensity of LCP and RCP in the hologram plane are not always equal to each other, additional freedom should be considered to control the intensity difference between LCP and RCP. In this paper, we use two lines of LCP and RCP to control the amplitude according to the interference effect of the two lines. The rotation angles are δ_+ and $\delta_+ + \Delta\delta_+$ for two LCP lines, and δ_- and $\delta_- + \Delta\delta_-$ for two RCP lines as shown in Figure 1A in the main text. The amplitude of the output LCP and RCP can be given as,

$$A_+^m = \left|e^{-i2\delta_+} + e^{-i2(\delta_+ + \Delta\delta_+)}\right|/2 = \sqrt{(1+\cos 2\Delta\delta_+)/2} \tag{14}$$

$$A_-^m = \left|e^{i2\delta_-} + e^{i2(\delta_- + \Delta\delta_-)}\right|/2 = \sqrt{(1+\cos 2\Delta\delta_-)/2} \tag{15}$$

which can be simplified as Eq. 2 in the main text. The total intensity for LCP and RCP in the metasurface plane can be described as,

$$I_\sigma^m = (A_\sigma^m)^2 \tag{16}$$

$$I^m = I_+^m + I_-^m \tag{17}$$

Since the intensity of LCP and RCP in the image plane can be calculated from Supplementary Eq. 1 as,

$$I_\sigma^f = \sum_{x,y=1}^{N_x,N_y}\left(a_\sigma^f(x^f,y^f)\right)^2 \tag{18}$$

where x^f and y^f represent the coordinate of the pixels in the image plane, N_x and N_y are the total pixel number in x^f and y^f direction. According to the Supplementary Eq. 14-18, and using the fact that $I_\sigma^m = I_\sigma^f$, we can get the relationship of $\Delta\delta_+$ and $\Delta\delta_-$ as,

$$\frac{1+\cos 2\Delta\delta_+}{1+\cos 2\Delta\delta_-} = \frac{\sum_{x,y=1}^{N_x,N_y}\left(a_+^f(x^f,y^f)\right)^2}{\sum_{x,y=1}^{N_x,N_y}\left(a_-^f(x^f,y^f)\right)^2} \tag{19}$$

There are three possibilities as discussed below:

(1) if the intensity $I_+^f = I_-^f$, we choose

$$\Delta\delta_+ = \Delta\delta_- = 0 \tag{20}$$

(2) if $I_+^f > I_-^f$, we keep $\Delta\delta_+ = 0$ and $\Delta\delta_-$ is calculated from Supplementary Eq. 19 as,

$$\begin{cases} \Delta\delta_+ = 0 \\ \Delta\delta_- = \mathrm{acos}\left(\frac{2\sum_{x,y=1}^{N_x,N_y}\left(a_-^f(x^f,y^f)\right)^2}{\sum_{x,y=1}^{N_x,N_y}\left(a_+^f(x^f,y^f)\right)^2} - 1\right)/2 \end{cases} \tag{21}$$

(3) if $I_+^f < I_-^f$, we keep $\Delta\delta_- = 0$ and $\Delta\delta_+$ is calculated from Supplementary Eq. 19 as,

$$\begin{cases} \Delta\delta_+ = \mathrm{acos}\left(\frac{2\sum_{x,y=1}^{N_x,N_y}\left(a_+^f(x^f,y^f)\right)^2}{\sum_{x,y=1}^{N_x,N_y}\left(a_-^f(x^f,y^f)\right)^2} - 1\right)/2 \\ \Delta\delta_- = 0 \end{cases} \tag{22}$$

Supplementary Eq. 20-22 can be simplified as,

$$\begin{cases} \Delta\delta_\sigma = 0, & if\ I_\sigma^f \geq I_{-\sigma}^f \\ \Delta\delta_\sigma = \mathrm{acos}\left(\frac{2\sum_{x,y=1}^{N_x,N_y}\left(a_\sigma^f(x^f,y^f)\right)^2}{\sum_{x,y=1}^{N_x,N_y}\left(a_{-\sigma}^f(x^f,y^f)\right)^2} - 1\right)/2, & if\ I_\sigma^f < I_{-\sigma}^f \end{cases} \tag{23}$$

which is presented in Eq. 4 in the main text.

Therefore, the final orientation angle is obtained from Supplementary Eq. 13 and 23.

Supplementary Note 3: Measurement of the polarization parameters

The optical setup of the polarization measurement is shown in Supplementary Figure 2. A quarter waveplate with its fast axis along the *x*-axis and a linear polarizer with the transmission axis rotated through an angle of θ with respect to the *x*-axis is placed before the holographic image. The intensity of the optical beam after the waveplate and linear polarizer is related to the Stokes parameters as [50, 51],

$$I(\theta, \phi) = \frac{1}{2}(S_0 + S_1 \cos 2\theta + S_2 \sin 2\theta \cos \phi - S_3 \sin 2\theta \sin \phi) \tag{24}$$

where θ is the rotation angle of the linear polarizer and ϕ is the phase of the waveplate. Firstly, we remove the waveplate and measure sequentially the intensity of the output light with the linear polarizer set at $\theta = 0°$, 45°, and 90°, respectively. Then the final (fourth) measurement is carried out with the quarter-waveplate ($\phi = 90°$) together with a linear polarizer set at $\theta = 45°$. From Supplementary Eq. 24, we can get,

$$I(0°, 0°) = \frac{1}{2}(S_0 + S_1) \tag{25}$$

$$I(45°, 0°) = \frac{1}{2}(S_0 + S_2) \tag{26}$$

$$I(90°, 0°) = \frac{1}{2}(S_0 - S_1) \tag{27}$$

$$I(45°, 90°) = \frac{1}{2}(S_0 - S_3) \tag{28}$$

Therefore, the Stokes parameter is given by,

$$S_0 = I(0°, 0°) + I(90°, 0°) \tag{29}$$

$$S_1 = I(0°, 0°) - I(90°, 0°) \tag{30}$$

$$S_2 = 2I(45°, 0°) - I(0°, 0°) - I(90°, 0°) \tag{31}$$

$$S_3 = I(0°, 0°) + I(90°, 0°) - 2I(45°, 90°) \tag{32}$$

The azimuth angle and ellipticity angle can be extracted from the Stokes parameter as,

$$\psi = \frac{1}{2}\tan^{-1}\left(\frac{S_2}{S_1}\right) \qquad (0 < \psi < \pi) \tag{33}$$

$$\chi = \frac{1}{2}\sin^{-1}\left(\frac{S_3}{S_0}\right) \qquad \left(-\frac{\pi}{4} < \chi < \frac{\pi}{4}\right) \tag{34}$$

Therefore, from Supplementary Eq. 29-34, we can calculate azimuth angle and ellipticity angles as,

$$\psi = \frac{1}{2}\tan^{-1}\left(\frac{2I(45°,0°)-I(0°,0°)-I(90°,0°)}{I(0°,0°)-I(90°,0°)}\right) \qquad (0 < \psi < \pi) \tag{35}$$

$$\chi = \frac{1}{2}\sin^{-1}\left(\frac{I(0°,0°)+I(90°,0°)-2I(45°,90°)}{I(0°,0°)+I(90°,0°)}\right) \qquad \left(-\frac{\pi}{4} < \chi < \frac{\pi}{4}\right) \tag{36}$$

which is given in the main text.

Supplementary Note 4: Matlab code for the modified Gerchberg–Saxton algorithm

```
% Author: Song Qinghua
% e-mail: songqinghua150@163.com
% -----------------------------------------------------

% -----------------------------------------------------
% Paper:  "Broadband decoupling of intensity and polarization
with vectorial Fourier metasurfaces"
% Journal: Nature Communications
% !!!Please cite this paper if this code helps you. Thanks!!!
% -----------------------------------------------------

clc; clear all; close all;
resx=200; resy=200; % resolution of images
input_intensity=ones(resy,resx); % input intensity profile
Target_amp = imread('DataAmplitude.png'); % amplitude profile in
the far field
Target_azi = imread('DataAzimuth.png'); % azimuth in the far
field
Target_elli = imread('DataEllipticity.png'); % ellipticity in the
far field
Target_amp=double(Target_amp);
Target_azi=double(Target_azi)/255*pi/2-pi/4;
Target_elli=double(Target_elli)/255*pi/4-pi/8;

Amp = Target_amp;
azimuth = Target_azi;
ellipticity = Target_elli;

alphi = 2*azimuth; % phase difference between RCP and LCP;
aL = ((Amp.^2-Amp.^2.*sin(2.*ellipticity))/2).^0.5; % amplitude
of LCP;
aR = ((Amp.^2+Amp.^2.*sin(2.*ellipticity))/2).^0.5; % amplitude
of RCP;

results_int_RCP_sum = sum(sum(aR.^2)); % total intensity of RCP;
results_int_LCP_sum = sum(sum(aL.^2)); % total intensity of LCP;

AR =
fftshift(ifft2(fftshift(aR.*exp(1i.*rand(resy,resx)*2*pi))));
AL =
fftshift(ifft2(fftshift(aL.*exp(1i.*rand(resy,resx)*2*pi))));

iteration_num = 101;

for i=1:iteration_num
    if i==iteration_num-1
            AAR=AR;
            AAL=AL;
    end
```

```
    if mod(i,2)==1
        BR = abs(input_intensity) .* exp(1i*angle(AR));
        CR = fftshift(fft2(fftshift(BR)));
        DR = aR .* exp(1i*angle(CR));
        AR = fftshift(ifft2(fftshift(DR)));
        BL = abs(input_intensity) .* exp(1i*angle(AL));
        CL = fftshift(fft2(fftshift(BL)));
        DL = aL .* exp(1i*(angle(CR)-alphi));
        AL = fftshift(ifft2(fftshift(DL)));
    else if mod(i,2)==0
        BL = abs(input_intensity) .* exp(1i*angle(AL));
        CL = fftshift(fft2(fftshift(BL)));
        DL = aL .* exp(1i*(angle(CL)));
        AL = fftshift(ifft2(fftshift(DL)));
        BR = abs(input_intensity) .* exp(1i*angle(AR));
        CR = fftshift(fft2(fftshift(BR)));
        DR = aR .* exp(1i*(angle(CL)+alphi));
        AR = fftshift(ifft2(fftshift(DR)));
        end
    end
end

    results_amp_RCP = abs(CR).*results_int_RCP_sum^0.5;
    results_amp_LCP = abs(CL).*results_int_LCP_sum^0.5;
    results_phase_RCP = angle(CR);
    results_phase_LCP = angle(CL);
    results_alphi = mod(results_phase_RCP-
results_phase_LCP,2*pi);
    results_int_contrast =
results_int_RCP_sum/results_int_LCP_sum;
    results_amp_RCPplusLCP =
(results_amp_RCP.^2+results_amp_LCP.^2).^0.5;
    results_azimuth = results_alphi/2;
    results_ellipticity = asin((results_amp_RCP.^2-
results_amp_LCP.^2)./(results_amp_RCP.^2+results_amp_LCP.^2))/2;

    for j=1:resx
        for jj=1:resy
            if results_azimuth(j,jj)>pi/2
                results_azimuth(j,jj)=results_azimuth(j,jj)-pi;
            end
        end
    end

    figure
    subplot(2,2,1);
    imagesc(Amp);
    title('Original Amplitude')
    axis equal;axis tight;
    colormap Hot;
    subplot(2,2,2);
    imagesc(results_amp_RCPplusLCP)
```

```
title('Reconstructed Amplitude');
axis equal;axis tight;
subplot(2,2,3);
imagesc(results_amp_RCP)
title('Reconstructed RCP');
axis equal;axis tight;
subplot(2,2,4);
imagesc(results_amp_LCP)
title('Reconstructed LCP');
axis equal;axis tight;

figure
subplot(2,2,1);
imagesc(Target_azi/pi*180)
title('Original azimuth');
axis equal;axis tight;
colormap Hot;
caxis([-90 90]); set([colorbar],'fontsize',16);
subplot(2,2,2);
imagesc(Target_elli/pi*180)
title('Original ellipticity');
axis equal;axis tight;
colormap Hot;
caxis([-45 45]); set([colorbar],'fontsize',16);
subplot(2,2,3);
imagesc(results_azimuth/pi*180)
title('Reconstructed azimuth');
axis equal;axis tight;
colormap Hot;
caxis([-90 90]); set([colorbar],'fontsize',16);
subplot(2,2,4);
imagesc(results_ellipticity/pi*180)
title('Reconstructed ellipticity');
axis equal;axis tight;
colormap Hot;
caxis([-45 45]); set([colorbar],'fontsize',16);

%% Holographic phase profile
phase_RCP = angle(AAR);
phase_LCP = angle(AAL);
```

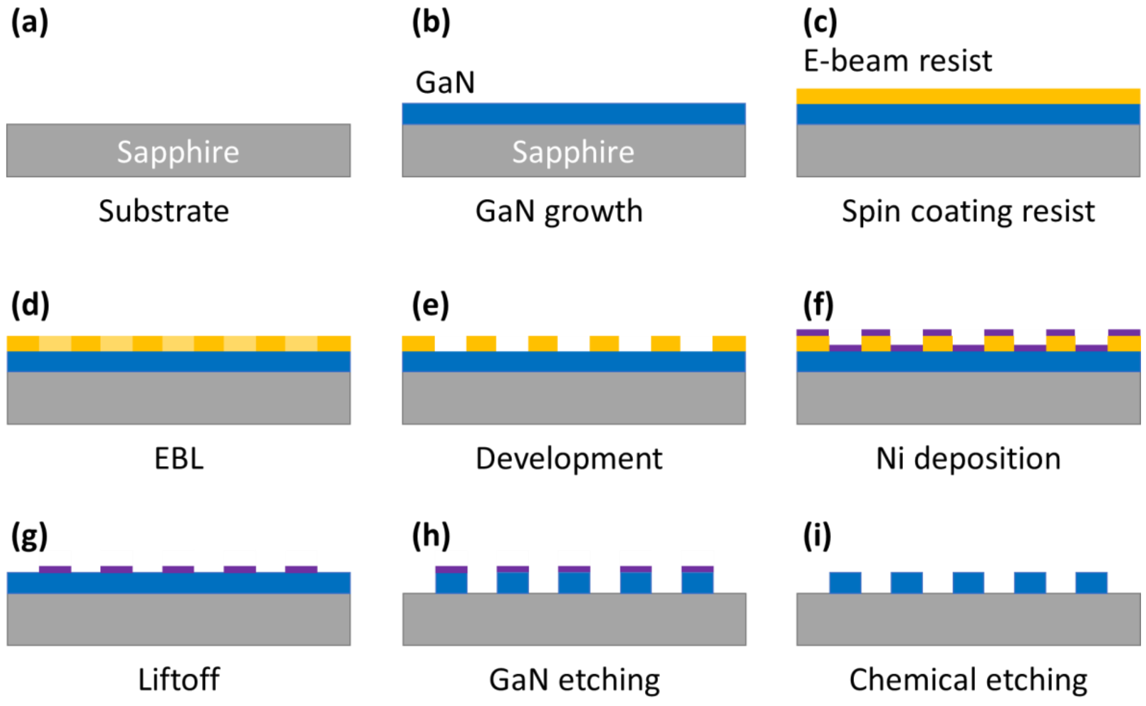

Supplementary Figure 1. Fabrication processes of metasurface. (**A**) A double-side polished c-plan sapphire is used as the substrate. (**B**) GaN thin-film with 1 μm thickness is grown on sapphire substrate. (**C**) Spin coated with PMMA resist. (**D**) Exposure using electron beam lithography. (**E**) Development in 3:1 IPA:MIBK solution. (**F**) Nickel deposition with 50 nm using E-beam evaporation. (**G**) Liftoff process in acetone. (**H**) GaN etching by reactive ion etching. (**I**) Chemical etching in 1:1 H_2O_2: H_2SO_4 solution to remove the nickel. PMMA: poly(methyl methacrylate). IPA: isopropyl alcohol. MIBK: methyl isobutyl ketone. EBL: e-beam lithography.

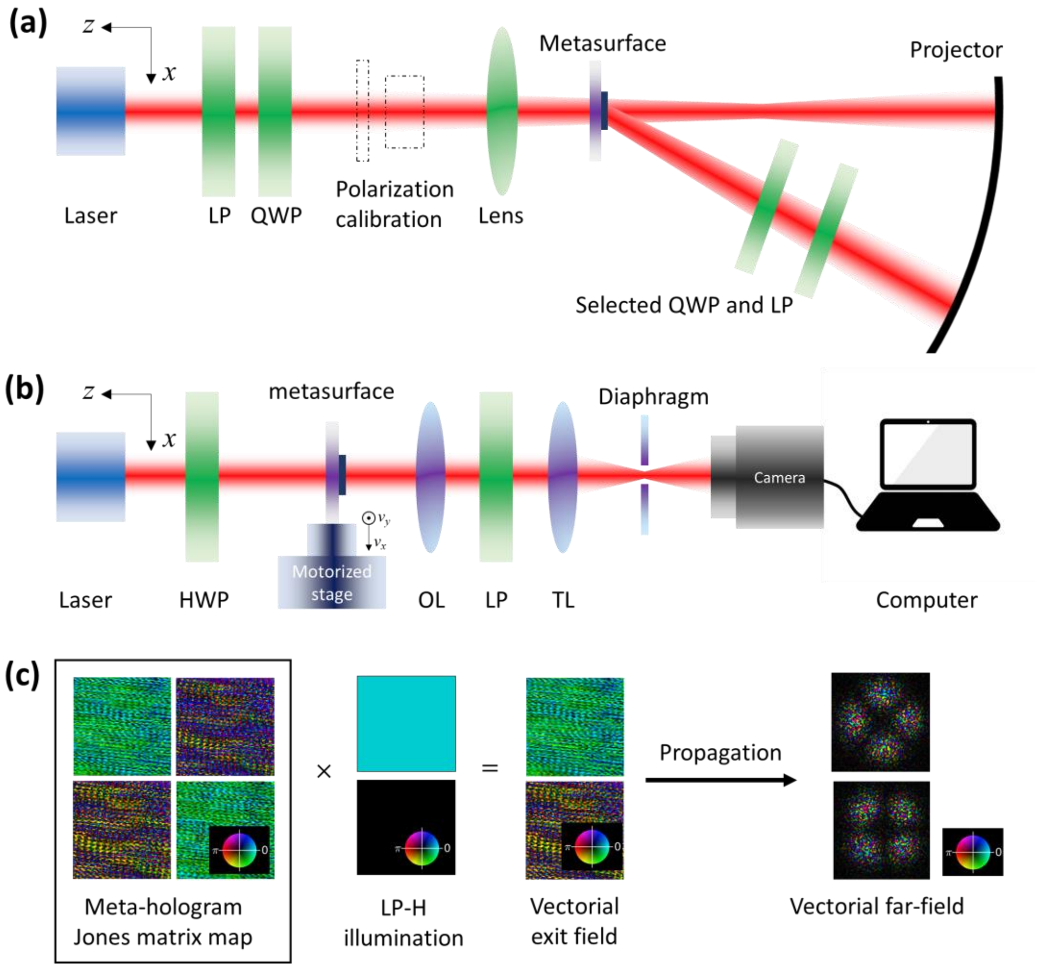

$$\begin{bmatrix} J_{xx} & J_{yx} \\ J_{xy} & J_{yy} \end{bmatrix} \times \begin{bmatrix} 1 \\ 0 \end{bmatrix} = \begin{bmatrix} E_x \\ E_y \end{bmatrix} \xrightarrow{\text{Fourier transform}} \begin{bmatrix} \hat{E}_x \\ \hat{E}_y \end{bmatrix}$$

Supplementary Figure 2. Measurement schemes for conventional polarimetry and vectorial ptychography. (**a**) Schematic of the conventional optical setup. A laser with wavelength of 635 nm is passing through a linear polarizer (LP) and a quarter waveplate (QWP). In order to avoid the birefringent effect of the sapphire substrate, we calibrate the polarization of the input light with a bare sapphire substrate to make sure the input light is linear polarized in horizontal direction by controlling the rotation angle of the previous LP and QWP. The polarization calibration components consist of a bare sapphire substrate with the same thickness of the metasurface substrate and a polarimeter for the measurement of the polarization as shown in the dash line, which are removed after the calibration. After calibration, the laser passes through a lens to weakly focus on the metasurface. A projector with a distance of 10 cm to the metasurface is placed to display the holographic images. Selected quarter waveplate and linear polarizer are placed before the projector to analyze the holographic images and measure the Stokes parameters. (**b**) Schematic of the ptychographic measurement optical setup. For a detailed description, see Ref 55 in the main text. (**c**) Modelling of the vectorial far-field from the Jones matrix map. LP: linear polarizer. QWP: quarter waveplate. HWP: half waveplate. OL: objective lens. TL: tube lens. LP-H: linear polarization in horizontal.

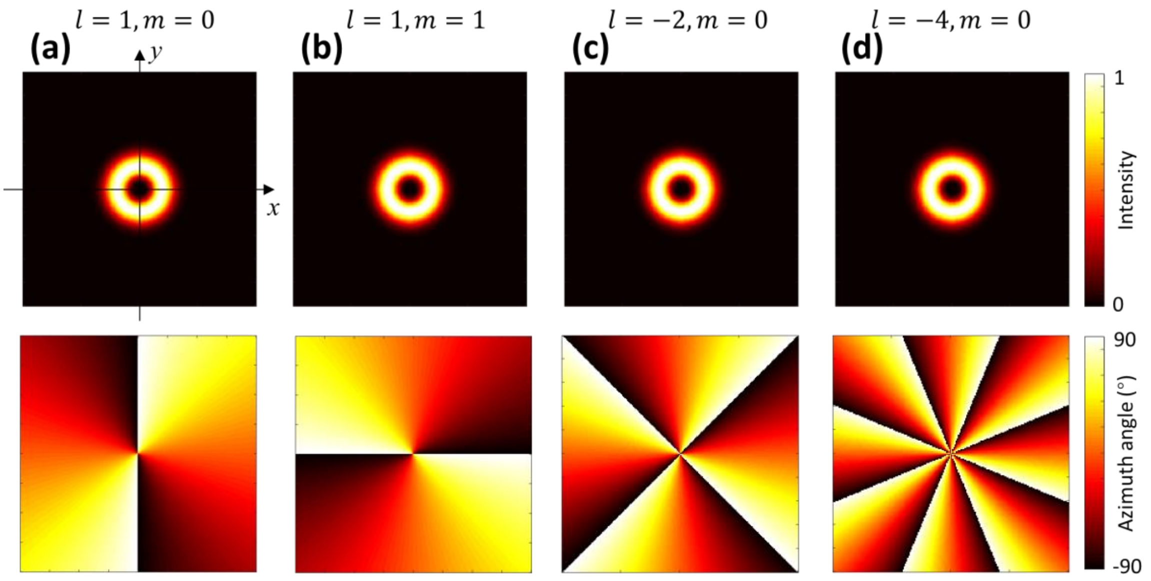

Supplementary Figure 3. Design of field profile mimicking the cylindrical vector beams. The field profile design with (**a**) *l* = 1, *m* = 0, (**b**) *l* = 1, *m* = 1, (**c**) *l* = -2, *m* = 0, (**d**) *l* = -4, *m* = 0. Top row: intensity profile. Bottom row: azimuth angle profile. The ellipticity angle of all of the designs are zero. Note that with respect to CV beams, the far-field phase distribution is not imposed.

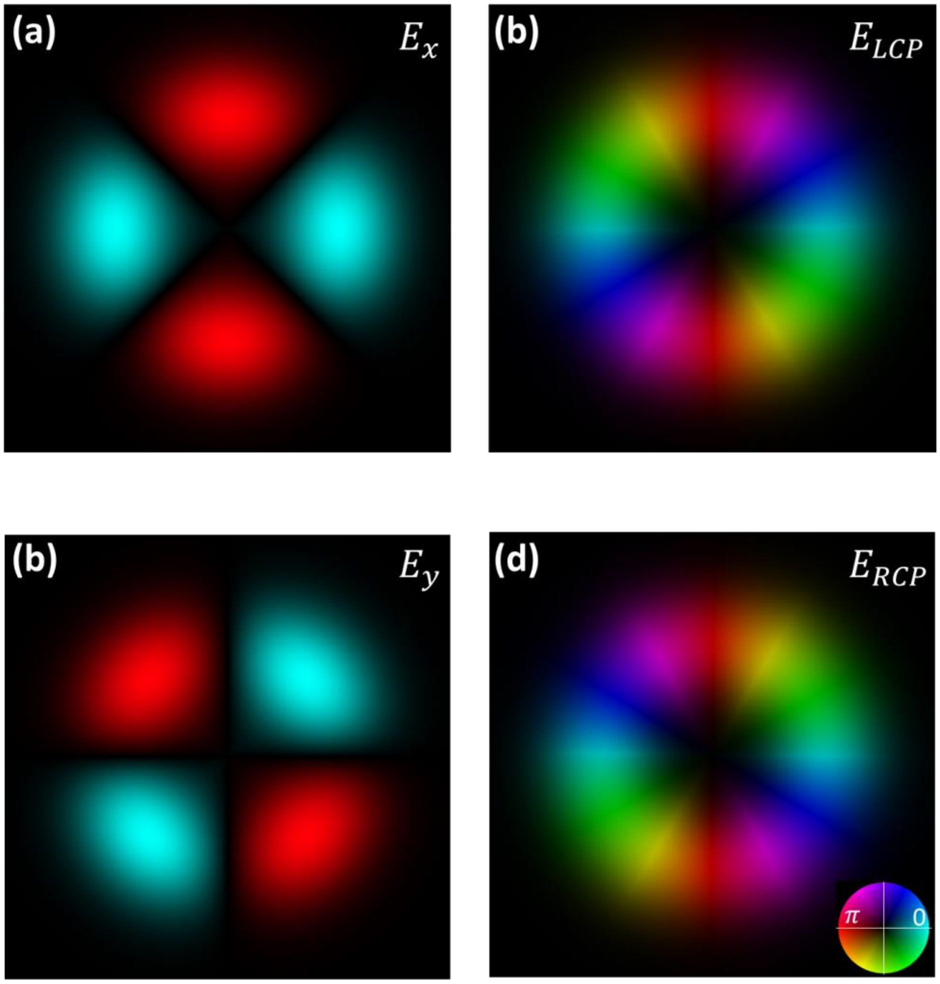

Supplementary Figure 4. Conventional CV beams. Complex amplitude in (**a**) *x*-polarization, (**b**) *y*-polarization, (**c**) LCP, and (**d**) RCP. The inset figure in (d) is the color bar with phase encoded as hue and amplitude as brightness.

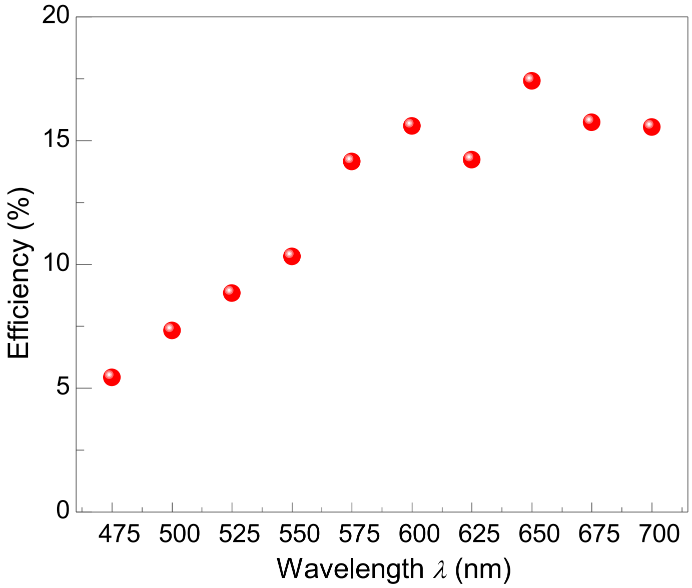

Supplementary Figure 5. Measured efficiency of the metasurface presented in Fig. 6 in the main text with the wavelength ranging from 475 nm to 700 nm.

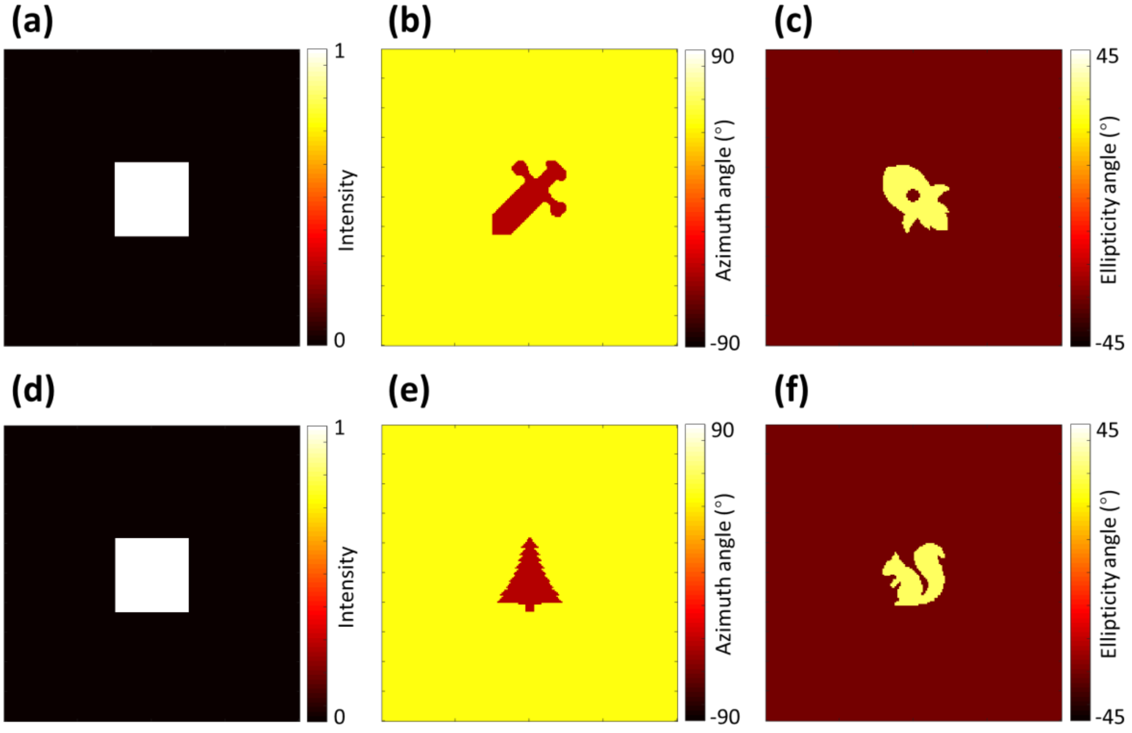

Supplementary Figure 6. Design of the metasurface for optical encryption. (**a**) – (**c**) Metasurface design 1 with uniformly distributed intensity profile in (**a**), a "Blade" image in (**b**) and a "Rocket" image in (**c**). (**d**) – (**f**) Metasurface design 2 with uniformly distributed intensity profile in (**d**), a "Tree" image in (**e**) and a "Squirrel" image in (**f**).

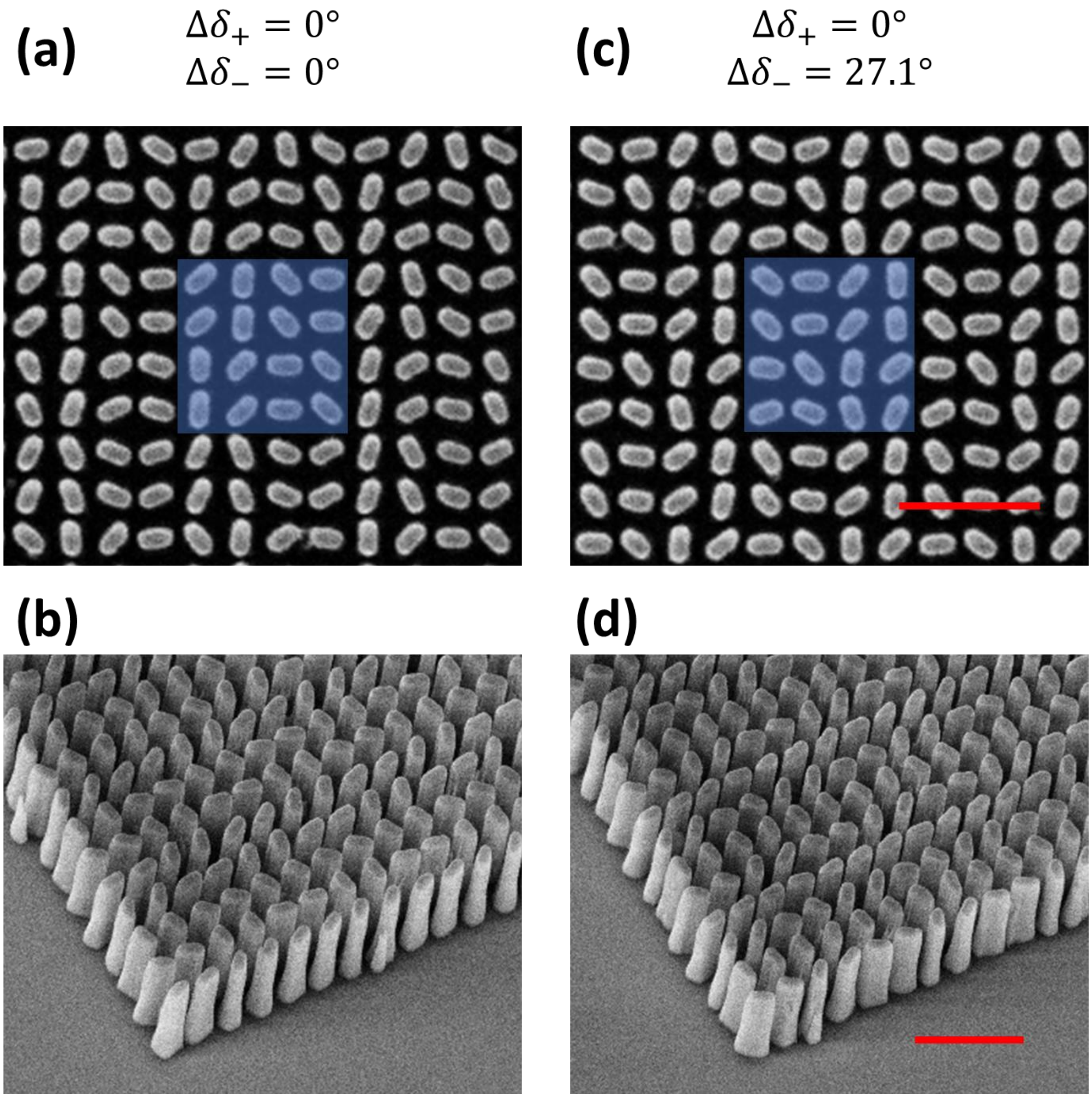

Supplementary Figure 7. Fabricated results of metasurface design 1 and design 2 for the optical encryption. (**a**) Top view and (**b**) tilt view of metasurface design 1 with rotation angle difference of $\Delta\delta_{\pm} = 0°$. (**c**) Top view and (**d**) tilt view of metasurface design 2 with rotation angle difference of $\Delta\delta_+ = 0°$ and $\Delta\delta_+ = 27.1°$. The red scale bar represents 1μm.

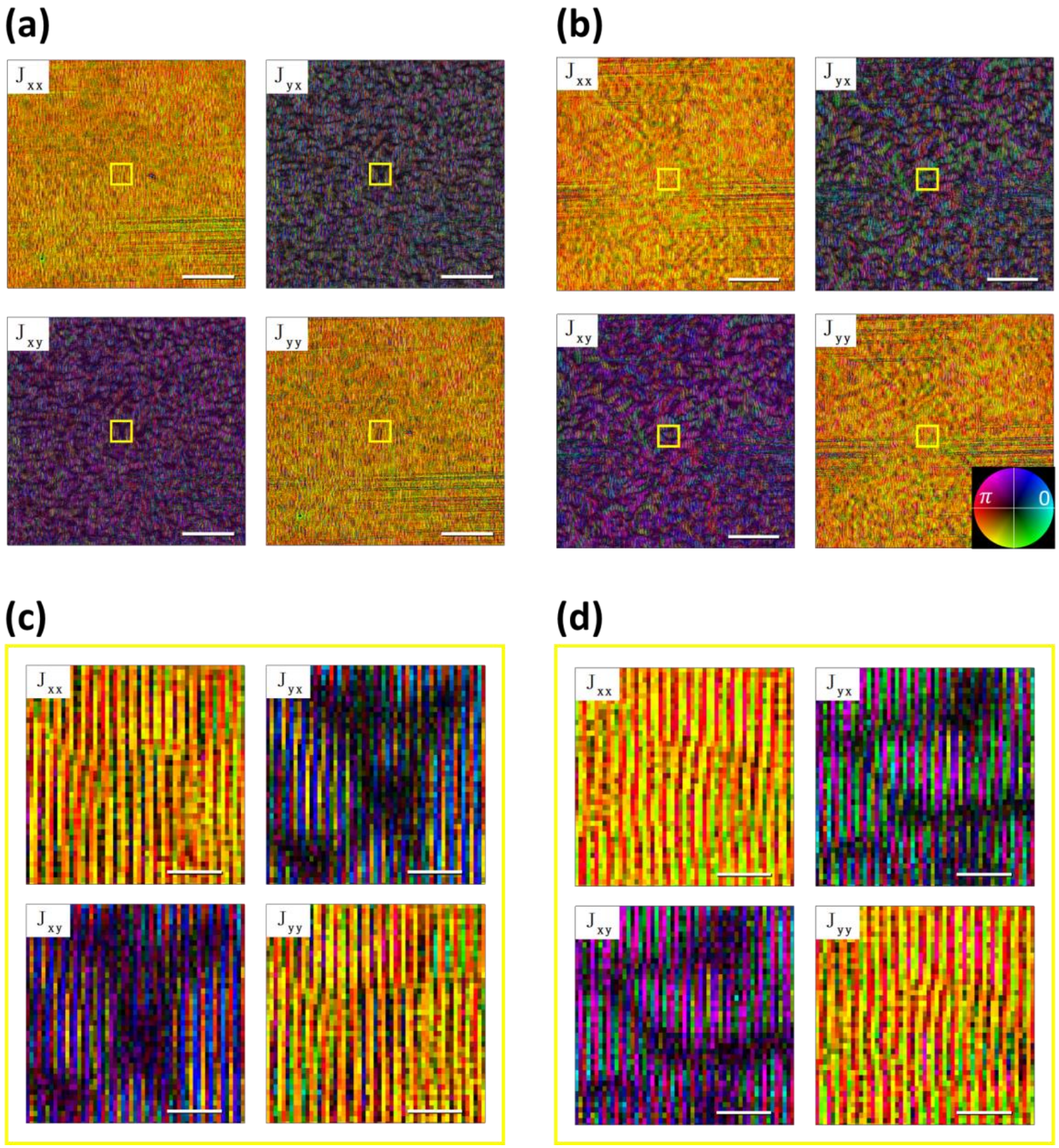

Supplementary Figure 8. Retrieved Jones matrix through ptychographic measurement. Measured Jones matrix of (**a**) Metasurface 1 and (**b**) Metasurface 2. The enlarged images of the central rectangle area are shown in (**c**) and (**d**). The inset figure in (**b**) is the color bar with phase encoded as hue and amplitude as brightness. The scale bar in (**a**) and (**b**) is 50 μm. The scale bar in (**a**) and (**b**) is 5 μm.

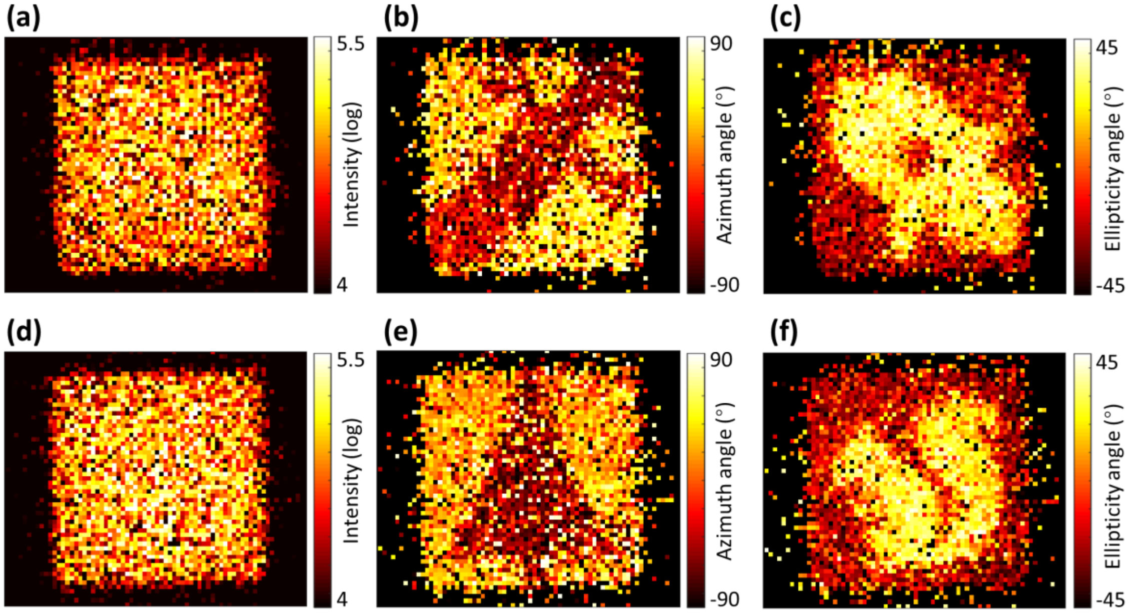

Supplementary Figure 9. Measurement results of vectorial encoded images using ptychographic measurement. (**a**) and (**d**) Retrieved intensity, (**b**) and (**e**) azimuth angle, (**c**) and (**f**) ellipticity angle of the polarization. A uniformly distributed intensity profile is shown in (**a**) and (**d**), a "Blade", "Rocket", "Tree" and "Squirrel" images are shown in (**b**), (**c**), (**e**) and (**f**). The measurement using conventional optical setup and ptychographic setup agree with the design.
